# Difficult Airway Management After Refusal of Awake Fibreoptic Intubation in a Giant Facial Plexiform Neurofibroma

**DOI:** 10.7759/cureus.113065

**Published:** 2026-07-21

**Authors:** Rajesh Kumar, Soumya Singh, Supreet Kaur, Shashi Kant, Sanjay Kumar

**Affiliations:** 1 Anaesthesiology, All India Institute of Medical Sciences, Patna, Patna, IND; 2 Anaesthesiology, Pain Medicine and Critical Care, All India Institute of Medical Sciences, New Delhi, New Delhi, IND

**Keywords:** difficult airway, fibreoptic intubation, giant facial neurofibroma, i-gel, neurofibromatosis type 1, plexiform neurofibroma, staged debulking, supraglottic airway

## Abstract

Neurofibromatosis type 1 (NF1) is an autosomal dominant disorder characterised by café-au-lait macules, cutaneous neurofibromas, and plexiform neurofibromas, which may extensively infiltrate surrounding tissues and create significant airway challenges during anaesthesia. We report the anaesthetic management of a 33-year-old man with a giant left-sided facial plexiform neurofibroma involving the glabella, eyelid, cheek, lips, chin, and hard palate undergoing his third staged debulking surgery. Although awake fibreoptic intubation was recommended, the patient declined the procedure. A bridging airway strategy was therefore adopted using an i-gel (Intersurgical, Wokingham, UK) supraglottic airway to establish ventilation, followed by fibreoptic-guided tracheal intubation through the device. Anaesthesia and surgery were completed uneventfully with stable haemodynamics, and the patient was extubated awake before transfer to the intensive care unit. This case demonstrates that an i-gel-assisted fibreoptic intubation technique is a safe and effective alternative when awake fibreoptic intubation is refused in patients with anticipated difficult airways due to giant facial plexiform neurofibromas.

## Introduction

Neurofibromatosis type 1 (NF1) is an autosomal dominant, multisystem disorder, recently redefined by international consensus criteria that incorporate genetic testing alongside the classic cutaneous and skeletal features [1]. Plexiform neurofibromas, an uncommon but characteristic NF1 manifestation, can involve the face, oropharynx, and larynx, producing tissue distortion that makes both bag-mask ventilation and laryngoscopy unpredictable [2,3]. Surgical debulking remains the mainstay for established, disfiguring craniofacial lesions, although mitogen-activated protein kinase kinase (MEK) inhibitors have recently changed the picture for symptomatic, inoperable plexiform disease and are reshaping how "limited" the treatment options are now considered [4]. Awake fibreoptic intubation is generally regarded as the safest approach when a difficult airway is anticipated, but it depends on patient cooperation, which is not always obtainable. We report a case of staged facial plexiform neurofibroma debulking in which an anticipated difficult airway was managed after induction, using a supraglottic device as a conduit for flexible scope intubation, when the patient declined an awake approach.

## Case presentation

**Figure 1:**
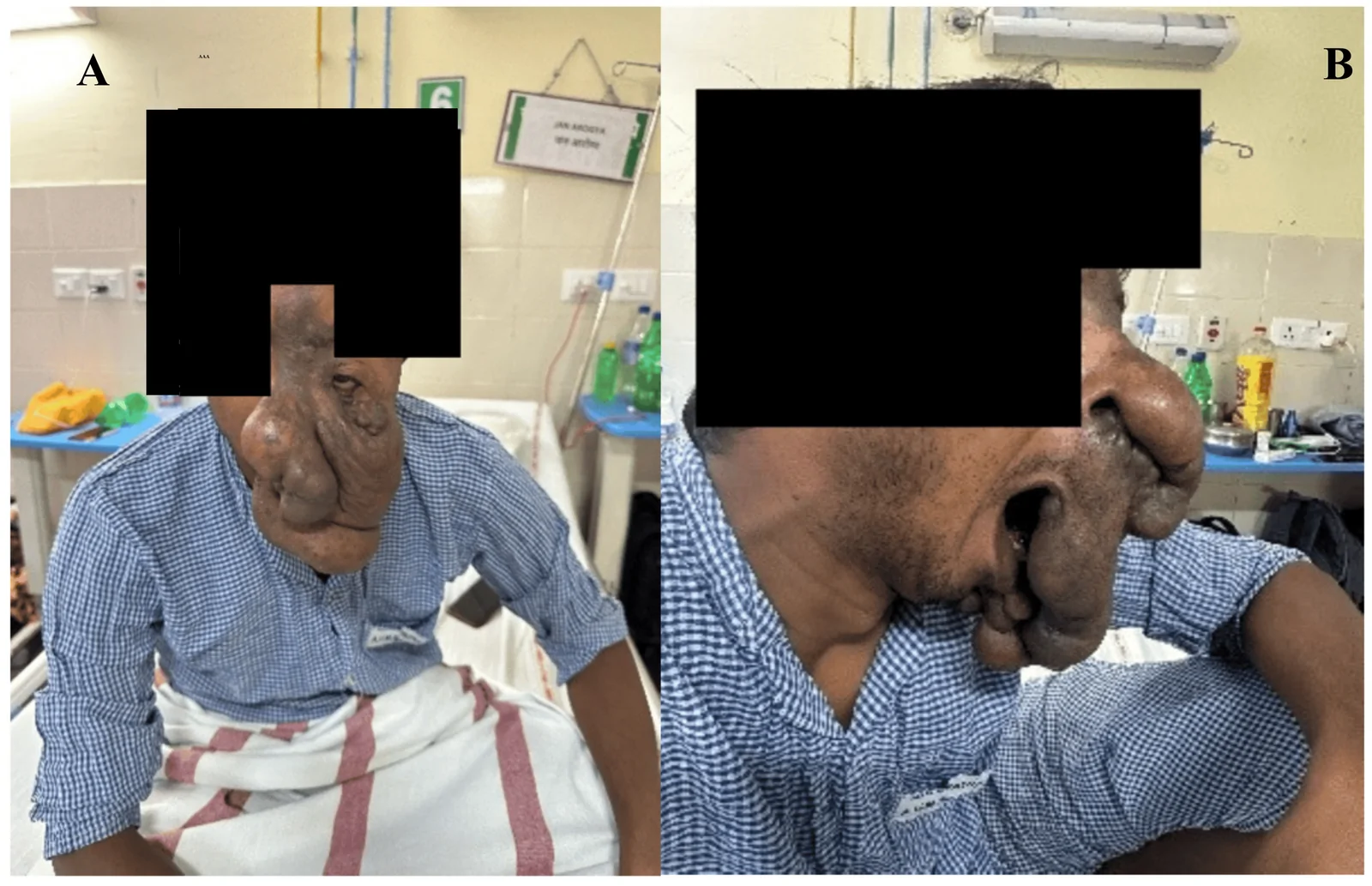
Preoperative clinical photographs of a giant left-sided facial plexiform neurofibroma. (A) Frontal view demonstrating the extensive involvement of the glabella, upper eyelid, nose, cheek, lips, and chin with marked facial asymmetry. (B) Left lateral view showing the extent of the tumour involving the nose, upper lip, cheek, and chin.

A 33-year-old man presented with a progressively enlarging giant swelling over the left side of his face since childhood. The swelling involved the glabella, upper eyelid, root of the nose, nose, left cheek, left half of the mouth, and chin, causing marked facial asymmetry (Figure 1).

Intraoral examination revealed a globulated mass involving the hard palate. He was scheduled for staged debulking surgery, having previously undergone similar procedures in 2008 and 2009.

Internal medicine consultation was taken to rule out other neurofibromatosis-related comorbidities. He had no associated comorbidities. Pre-anaesthetic evaluation showed no abnormalities on physical examination or routine laboratory investigations. Airway assessment revealed a mouth opening of more than three fingerbreadths, normal neck movements, and absent upper teeth. An otorhinolaryngology consultation confirmed a patent airway. Although awake fibreoptic intubation was advised, the patient declined the procedure despite counselling. Difficult bag-mask ventilation was anticipated because of the extensive facial deformity. Therefore, i-gel (Intersurgical, Wokingham, UK) insertion after induction was planned as a conduit for fibreoptic-guided intubation, and the surgical team was alerted regarding the possible need for emergency tracheostomy.

**Figure 2:**
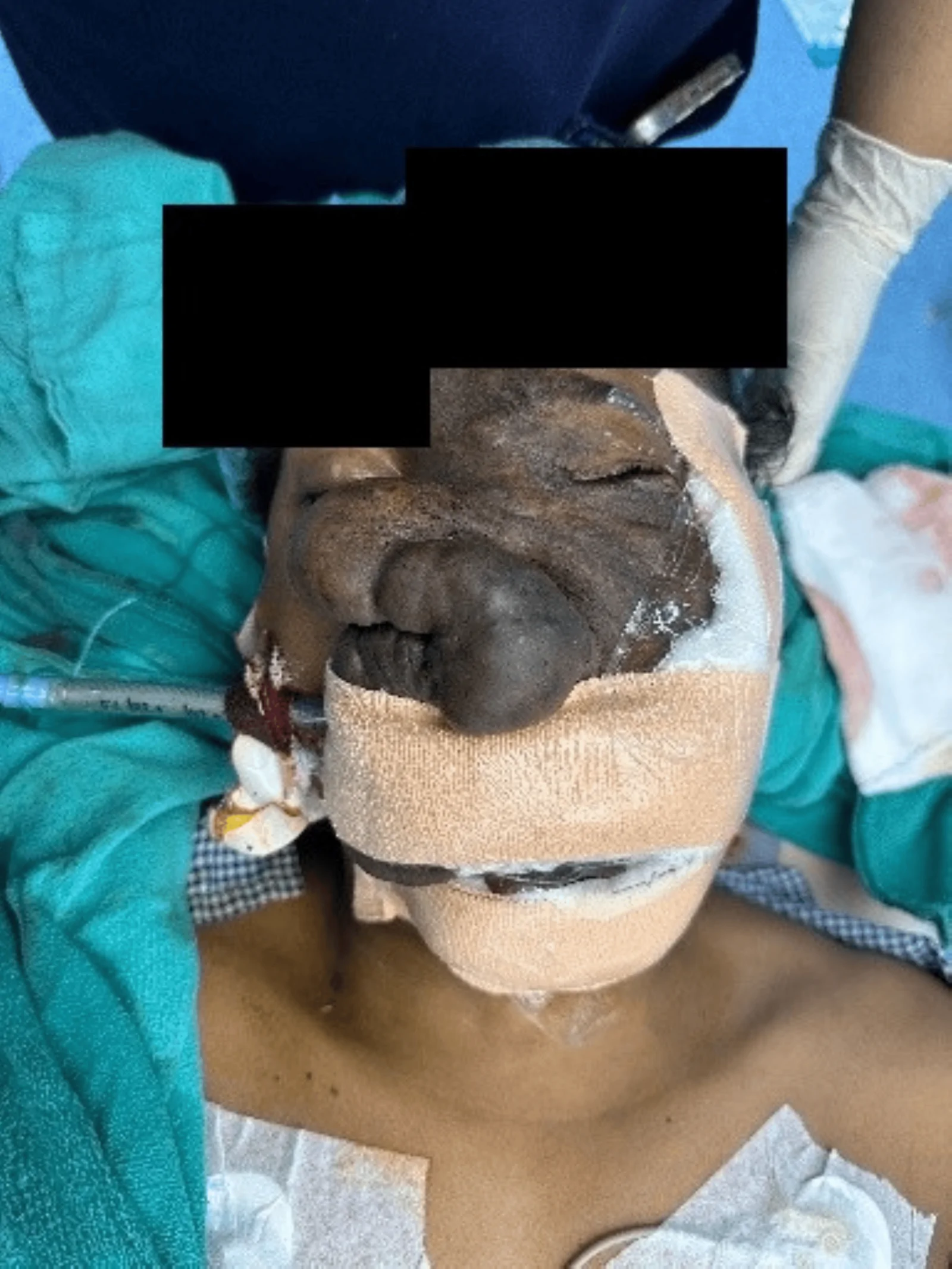
Successful oral endotracheal intubation following fibreoptic-guided airway management in a patient with a giant facial plexiform neurofibroma.

In the preoperative area, after confirming all documentation, the patient received 1 mg IV midazolam as an anxiolytic and was then shifted to the operation theatre. All standard American Society of Anesthesiologists (ASA) monitors were attached. Following the administration of fentanyl 100 μg and propofol 80 mg, initially a size 4 i-gel was inserted successfully, but there was a leak with the i-gel; then, the size was changed to 3. Once effective ventilation through the i-gel was confirmed, a flexible endoscope (Ambu® aScope™ 4 Broncho Slim 3.8/1.2, Ambu, Ballerup, Denmark) preloaded with an endotracheal tube (ETT) of size 6 was passed through the i-gel of size 3. After visualisation of the carina, the trachea was intubated under direct flexible endoscope guidance. Then, a tube exchanger (MediSafe® angulated tip airway exchange catheter with oxygen port, 15 Fr; MediSafe International, New Delhi, India) was put into the ETT, and the ETT was removed, but the tube exchanger was in place, an ETT of size 8 mm was railroaded over the tube exchanger, and the ETT was fixed at 22 cm. Correct tube placement was confirmed by bilateral air entry and capnography (Figure 2).

**Figure 3:**
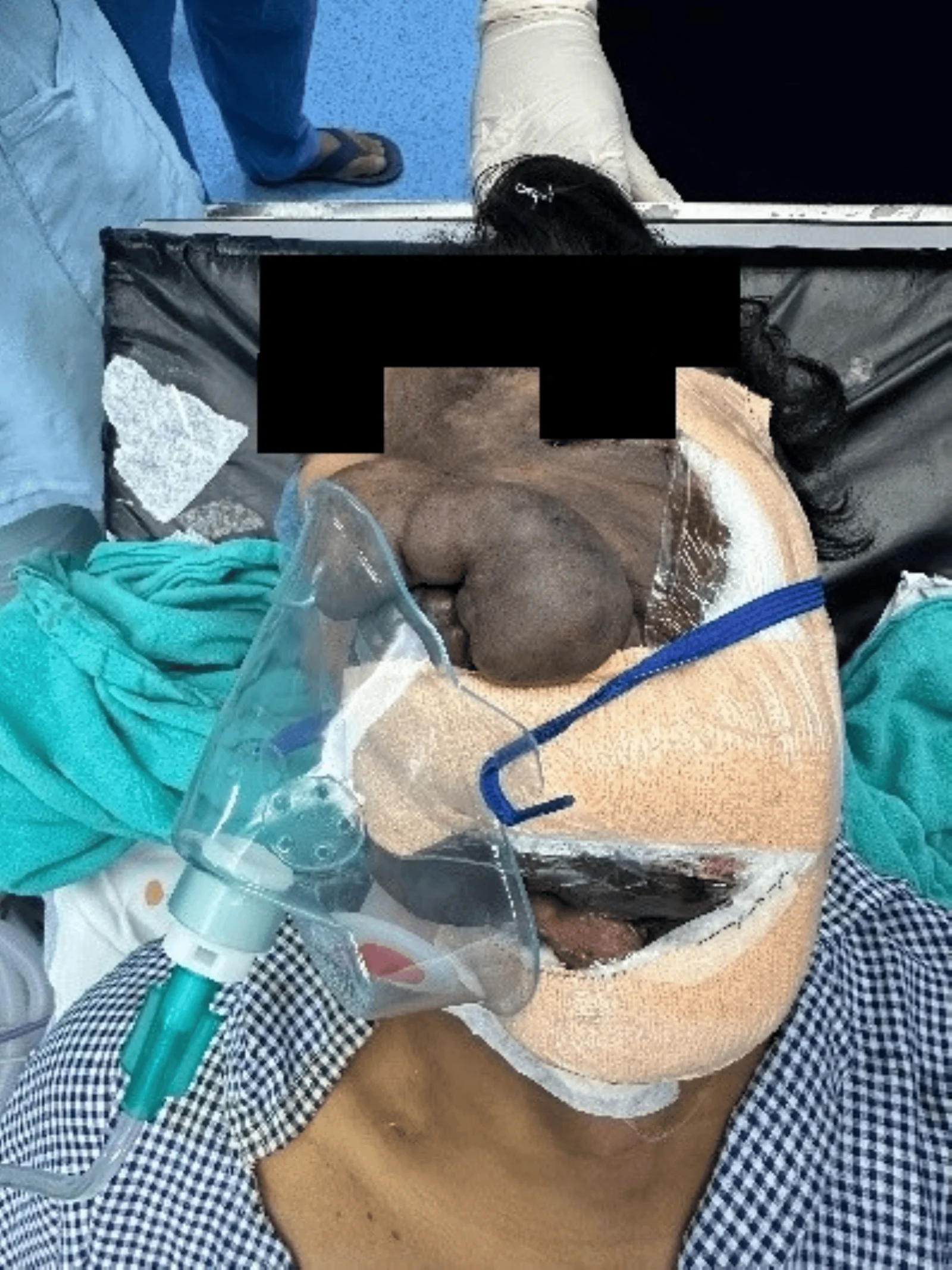
Immediate postoperative clinical photograph following staged debulking of the giant left-sided facial plexiform neurofibroma, demonstrating reduced tumour bulk and successful extubation with supplemental oxygen administered via face mask.

Anaesthesia was maintained with inhalational agents, atracurium, and controlled mechanical ventilation. A radial arterial catheter was inserted for invasive blood pressure monitoring. The surgery lasted approximately four hours and was uneventful. Neuromuscular blockade was reversed with neostigmine and glycopyrrolate. The patient was extubated after complete recovery of motor power and adequate spontaneous tidal volume. Supplemental oxygen was administered via face mask, and the patient was transferred to the post-anaesthesia care unit (PACU) for postoperative monitoring (Figure 3).

## Discussion

This case illustrates a pragmatic departure from the textbook approach. Awake fibreoptic intubation is widely considered the gold standard when facial or oropharyngeal plexiform neurofibroma threatens both mask ventilation and laryngoscopy, and several recent reports describe exactly that strategy for comparable facial disease [2,5]. Here, it was not available because the patient would not consent to it, which is a real and recurring constraint in this population rather than a rare exception.

The sequencing mattered as much as the technique. Visualising the glottis through the i-gel before administering a neuromuscular blocking agent allowed maintenance of the option to awaken the patient if airway access proved unsuccessful. Furthermore, ensuring that the surgical team was prepared for an emergency tracheostomy provided a predefined rescue strategy, consistent with the recommendations of the 2022 ASA Difficult Airway Guidelines [6].

One gap is worth naming rather than glossing over. NF1 carries a recognised, often silent, risk of pheochromocytoma, and case reports of intraoperative hypertensive crisis in exactly this setting are not rare curiosities [7]. The report states the patient had no associated comorbidities, but it is not clear whether this reflected biochemical screening or simply the absence of symptoms; an asymptomatic catecholamine-secreting tumour would not have announced itself before induction. Given the patient's surgical history and the four-hour debulking planned, a preoperative blood pressure trend or metanephrine check would have closed that gap rather than relying on intraoperative vigilance alone.

## Conclusions

In this patient, i-gel-assisted flexible scope intubation after induction secured the airway without desaturation, haemodynamic compromise, or rescue intervention across a four-hour procedure, demonstrating feasibility and safe conduct when awake fibreoptic intubation was refused, with preparedness for emergency tracheostomy forming the non-negotiable backstop that made proceeding after induction defensible. Giant facial plexiform neurofibromas demand more than airway planning: the full NF1 systemic burden, cardiovascular anomalies, occult pheochromocytoma, respiratory compromise from spinal or intrathoracic disease, and central nervous system (CNS) involvement require structured multidisciplinary evaluation completed before induction, not assumed normal in the absence of symptoms.
